# Cost-effectiveness of l-glutamine versus crizanlizumab for adults with sickle cell disease: model focused on reducing pain episode costs from Qatar’s healthcare perspective

**DOI:** 10.1177/20503121231224551

**Published:** 2024-05-06

**Authors:** Ahmad M Adel, Dina Abushanab, Daoud Al-Badriyeh, Anas Hamad, Awni Alshurafa, Mohamed A Yassin

**Affiliations:** 1 Pharmacy Department, National Center for Cancer Care and Research, Hamad Medical Corporation, Doha, Qatar; 2 College of Pharmacy, QU Health, Qatar University, Doha, Qatar; 3 Hematology Department, National Center for Cancer Care and Research, Hamad Medical Corporation, Doha, Qatar

**Keywords:** Sickle cell disease, crizanlizumab, l-glutamine, vaso-occlusive crisis, hemolytic anemia

## Abstract

**Objectives:**

Treatment options for preventing vaso-occlusive crises among sickle cell disease patients are on the rise, especially if hydroxyurea treatment has failed. This economic analysis is conducted to assess the comparative clinical effectiveness, safety, and acquisition cost of l-glutamine and crizanlizumab for older adolescents and adults (⩾16 years old) with sickle cell disease in Qatar, with an emphasis on treatment costs and acute pain crises.

**Methods:**

We conduct a decision-tree model, where we compare the clinical and economic outcomes of two novel Food and drug administration (FDA)-approved medications which are available in Qatar; l-glutamine and crizanlizumab over a time horizon of 1 year in a hypothetical cohort of adult sickle cell disease patients from a Qatar healthcare perspective. The main outcome is incremental cost per sickle cell disease-related acute pain crises averted. Model clinical parameters were derived from individual drug randomized trials, published literature, whereas cost parameters from Qatar healthcare payer system (2020–2021). A sensitivity analysis was carried out, and the study results were robust around model inputs. Costs were converted to 2020 US dollars.

**Results:**

Study results showed that both treatment modalities’ costs were the main driver of this analysis, with an average annual cost of the treatments per patient being $189,014 for crizanlizumab (5 mg/kg), $143,798 for crizanlizumab (2.5 mg/kg), and $74,323 for l-glutamine. The probability of no first-time sickle cell disease-related vaso-occlusive crises averted was 0.001/year for glutamine, 0.26/year for crizanlizumab (5 mg/kg), and 0.34/year for crizanlizumab (2.5 mg/kg). Lower dose crizanlizumab (2.5 mg/kg) dominated the higher one (5 mg/kg). The incremental cost-effectiveness ratio of crizanlizumab (2.5 mg/kg), when compared to l-glutamine was $81,265 per sickle cell disease-related vaso-occlusive crises averted. When comparing crizanlizumab (5 mg/kg) and l-glutamine, crizanlizumab (5 mg/kg) showed higher efficacy, yet the crizanlizumab incremental cost-effectiveness ratio was at $459,620 than l-glutamine.

**Conclusions:**

Crizanlizumab (2.5 mg/kg) may be a cost-effective intervention, yet it is not the approved dose for preventing vaso-occlusive crises in adolescents and adults with sickle cell disease. Crizanlizumab (5 mg/kg) was more cost-effective than the approved l-glutamine per sickle cell disease vaso-occlusive crisis prevented. Of note, we primarily focused on modeling acute vaso-occlusive pain, which limited our ability to consider other key outcomes in sickle cell disease.

## Introduction

Sickle cell disease (SCD) is a hereditary disease that is caused by autosomal recessive gene fault in the beta (β) allele of the hemoglobin (Hb) gene. As a result, sickled cells are characterized by easy and abnormal hemolysis with resultant varying degrees of anemia.^1^ Globally, the incidence of SCD is estimated to reach 400,000 persons per year, and in the United States alone, for example, the prevalence estimation is approximately 100,000 patients.^2^ Possible clinical presentations of SCD may come from different pathophysiologic mechanisms; the disfiguration of the RBC with subsequent loss of function can lead to vascular occlusion and a short lifetime of these RBCs that leads to hemolysis. The consequence of function loss is a vascular blockage and can cause vascular lesions.^3^ The most severe and serious manifestation of SCD is the recurrent acute pain, or better known as vaso-occlusive crisis (VOC).^4,5^ Additionally, other clinical manifestations that SCD patients may show are acute complications such as acute chest syndrome (ACS), recurrent infections, kidney necrosis, and stroke.^6^ Such complications may affect multiple organs and can result in early death.^6^ Acute pain crisis is another common complication of SCD and is usually managed with pain medications, especially opioids.^6,7^

In terms of SCD management, there is no universal curative treatment for SCD. Nonetheless, few medications are available for its symptoms and complication management. Of these medications in the market, hydroxyurea was the first approved medication for SCD management.^8^ Later, other drugs were approved and are indicated for the prevention of VOC, namely l-glutamine, crizanlizumab, and voxelotor.^9–11^ By cost, hydroxyurea is considered to be the cheapest option among all of these drugs, where healthcare costs for patient on hydroxyurea were $9450, compared with $13,716 with those who did not receive this treatment, in a 2-year study in the United States.^12^ However, hydroxyurea is usually under prescribed, and compliance to its administration is poor.^12^ It is worth mentioning that blood transfusions can also be used as a treatment modality in patients with SCD, but they carry the risk of high levels of iron in the blood, which may cause organ damage in long-term use.^13^

In relation to treatment costs, SCD management costs are high, with an estimated economic burden of $2.98 billion per year in the United States, with approximately 57% due to inpatient-based costs, 38% incurred by outpatient-based costs, and a remaining 5% as an out-of-pocket cost.^14^ Furthermore, the minimum treatment options, added to the stigma caused by the continuous need for pain management, make coping with SCD problematic.^15^

Of the drugs mentioned, two new therapies have become available for patients with SCD in the state of Qatar, l-glutamine and crizanlizumab. The management of SCD in Qatar follows that of American Society of Hematology.^7^ However, the clinical and economic impact of these treatments on preventing first pain crisis of SCD has never been compared in literature. Therefore, this assessment sought to evaluate the cost-effectiveness of crizanlizumab versus l-glutamine in preventing the first pain crisis among patients with SCD in Qatar.

## Methods

### Model structure

Clinical data were abstracted from landmark randomized controlled trials (RCTs), based on which drugs were granted approval. Additionally, treatment modalities included in data analysis has input from sickle cell treatment guidelines at the National Center for Cancer Care and Research (NCCCR) in Qatar to reflect the current practice in the country.

**Figure 1.**
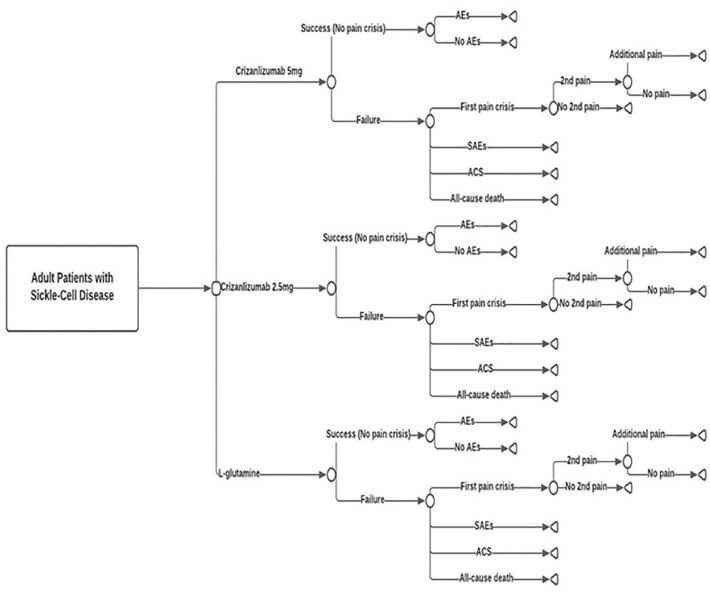
A schematic representation of the decision analysis model of study drugs.

A conventional decision-tree model was structured to generate the clinical pathways followed by SCD patients (Figure 1). The model alternatives were three possible treatment strategies: crizanlizumab (5 mg/kg), crizanlizumab (2.5 mg/kg), and l-glutamine. To note, while the 5 mg/kg is an approved dose for the crizanlizumab, the 2.5 mg/kg is not. The inclusion of 2.5 mg/kg is to reflect the practice used in SUSTAIN trial—from which input variables were derived.

Patients were initially differentiated based on success and failure. Success is to have no pain crisis, with/without adverse events (AEs). Failure is to develop pain crisis, ACS, or death. After the first pain crisis, a second may also develop. After the second pain, an additional pain crisis may develop. Second crisis is defined as the development of a second pain incident following a first pain episode. Based on the RCT sources of data, the duration of the model follow-up is 1 year. Figure 1 presents the decision-tree structure of study comparators.

Success is defined as having no pain crisis, death, ACS, or serious AEs. According to RCTs, a pain crisis is defined as pain resulting in receiving a parenterally administered narcotic or ketorolac in an emergency department or outpatient treatment center, or during hospitalization. Pain begins suddenly and lasts several hours to several days, can be mild to severe, and can last for any length of time.^16,17^ Death is defined as all-cause death. An AE is defined as any undesirable effect that is probably associated with the use of a medication in a patient.^18^ A serious AE, on the other hand, is defined as AE that may result in disability, hospitalization, or death.^18^ The ACS is defined as life-threatening and should be treated in a hospital. Signs and symptoms include chest pain, coughing, difficulty breathing, and fever.^17,18^ An expert panel of hematology consultants who are based at the NCCCR validated the structure of the model and its consequences.

### Sample size calculation

Unlike clinical trials, where sample size calculations are essential to ensure sufficient statistical power for detecting differences in primary endpoints, in our cost-effectiveness analysis, we simulate the progression of disease, treatment effects, and associated costs over time. These models allow for the exploration of various scenarios and assumptions, rendering traditional sample size calculations less applicable.

### Clinical inputs

Model probability inputs in relevance to events associated with crizanlizumab 5 mg and crizanlizumab 2.5 mg, including pain crises averted, AEs, ACS, and death were primarily obtained from the SUSTAIN trial by Ataga et al.^21^ Model clinical events, their probabilities, and sources of data can be seen in Table 1.

**Table 1.** Clinical outcomes probabilities of different modalities.

Treatments	Relative effect on acute pain crisis	Source
l-Glutamine	0.4503	NIIHARA trial^22^
Crizanlizumab 2.5 mg	0.5625	SUSTAIN trial^21^
Crizanlizumab 5 mg	0.5152	SUSTAIN trial^21^

The SUSTAIN trial is a phase 2, multicenter, randomized, placebo-controlled, double-blinded trial to assess the safety and efficacy of crizanlizumab (2.5 and 5 mg, administered 14 times, intravenously, over 1 year) with or without hydroxyurea in SCD patients—ClinicalTrials.gov Identifier: NCT03814746.^21^ The Niihara et al. study is a phase 3, multicenter, randomized, placebo-controlled, double-blinded, to evaluate the efficacy of l-glutamine (0.3 g/kg of body weight, administered orally twice daily).^22^ The studies by Ataga et al. and Niihara et al. are randomized trials that granted both medications the approval for their use in SCD, and they are consistent with NCCCR setting in relation to the used dose regimens of the study drugs. Important, is that the patient eligibility criteria in the main sources of data, the Ataga and Niihara et al. studies,^21,22^ were consistent with practices in NCCCR regarding the prevention of the first pain crisis in patients with a diagnosis of sickle cell anemia (SCA) (homozygous hemoglobin S) or sickle β0-thalassemia (HbSβ0-thalassemia) and having at least two pain crises documented during the previous year.

### Study perspective

The study was conducted from the NCCCR hospital’s perspective. The NCCCR is one of the hospitals under Hamad Medical Corporation (HMC), the main healthcare provider in Qatar, including 13 major specialized public hospitals.

### Cost inputs

Direct medical costs of resources consumed in the management of SCD were calculated (based on 2020–2021 prices). The cost data were obtained from the Finance and Costing Department of HMC, and included costs of medications acquisition by dispensing pharmacy, hospitalization, managing serious AEs, laboratory, and screening tests (Table 2). The medical care component of Qatar’s Consumer Price Index was used for cost inflation. All costs were expressed in 2021 Qatari Riyal (QAR) and then were converted to United States Dollar (USD). No discounting was applied as outcomes were not projected beyond a 1-year time horizon.

**Table 2.** Total cost of resources per patient.

Cost component	Crizanlizumab 5 mg (QAR)	Crizanlizumab 2.5 mg (QAR)	l-Glutamine (QAR)
Medications	382,981	191,490	143,538
Laboratory tests	1131.41	1131	1166
Screening tests	3428	3428	3428
Hospitalization, including management of pain crisis, hospital stay, intensive care unit (ICU) stay, medications used during hospitalization, laboratory tests performed during hospitalization	46,603	73,464	26,072
Management of serious AEs	2418	3069	7254

### Outcome measurement

The outcome of the study was the incremental cost-effectiveness ratio (ICER) in terms of QAR per additional case of pain crisis avoided. The outcome endpoint was basically the annual rate of pain crises, defined as the development of acute incidents of pain that resulted in either an urgent medical visit to the hospital or treatment with oral or parenteral narcotic agents or with a parenteral nonsteroidal anti-inflammatory drug (NSAID). Of note, no crisis means that patients finish the 1-year study with no pain episodes. Cost-effectiveness was determined based on a willingness-to-pay threshold of USD 150,000 (547,500 QAR) per outcome.

### Sensitivity analysis

A one-way sensitivity analysis was first conducted by assigning a ±15% uncertainty range to the cost of medications, using a triangular type of distribution. A probabilistic sensitivity analysis was conducted by introducing uncertainty to the base-case clinical events. A ±95% confidence interval (CI) uncertainty range of the base-case value was applied to clinical events using Trigen distribution. All sensitivity analyses were performed via the Monte Carlo simulation approach using @Risk-7.5^®^ (Palisade Corporation, Ithaca, NY, USA), with 5000 iterations.^24^

## Results

### Base-case analysis

The 5 mg crizanlizumab achieved a success rate of pain crisis averted of 0.5152 compared to 0.4503 with l-glutamine, with an incremental ICER of QAR 79,424 ($21,813) with 5 mg crizanlizumab per patient. Also, 2.5 mg crizanlizumab achieved a success rate of pain crisis averted of 0.5625 compared to 0.4503 with l-glutamine, with an ICER of QAR 73,226 ($20,111) with 2.5 mg crizanlizumab per patient. The 5 mg crizanlizumab and 2.5 mg crizanlizumab achieved a success rate of pain crisis averted of 0.5152 and 0.5625, respectively, with a cost saving of QAR 3552 ($976) with crizanlizumab 2.5 mg over crizanlizumab 5 mg per patient. This is, therefore, a dominance (higher effectiveness and lower cost) in favor of crizanlizumab 2.5 mg over crizanlizumab 5 mg.

Model pathway probabilities and their costs are as seen in Tables 3 and 4.

**Table 3.** Model pathway probabilities and their costs.

Outcomes	Crizanlizumab 5 mg			Crizanlizumab 2.5 mg			l-Glutamine		
	Pathway probabilities	Total cost (QAR)	Weighted cost	Pathway probabilities	Total cost (QAR)	Weighted cost	Pathway probabilities	Total cost (QAR)	Weighted cost
Success with AEs	0.4431	392,508	173,909	0.4894	200,593	98,165	0.4394	156,123	68,606
Success without AEs	0.0721	390,090	28,136	0.0731	197,524	14,444	0.0109	148,869	1618
Failure due to first pain crisis followed by second pain crisis followed by additional pain crisis	0.0150	1,249,771	18,729	0.0283	729,672	20,637	0.0072	493,509	3575
Failure due to first pain crisis followed by second pain crisis followed by no additional pain crisis	0.1991	818,850	163,034	0.1534	462,729	71,004	0.1724	317,690	54,756
Failure due to first pain crisis followed by no second pain crisis	0.2319	390,346	90,532	0.2409	198,856	47,904	0.2657	149,126	39,616
Failure due to ACS	0	434,313	—	—	269,683	—	0.0852	174,374	14,855
Failure due to death	0.0296	390,090	11,536	0.0154	197,524	3042	0.0131	148,869	1944
ICER	Crizanlizumab 5 mg versus crizanlizumab 2.5 mg: dominance, with QAR 3552 cost savings Crizanlizumab 5 mg versus l-glutamine: QAR 79,424 Crizanlizumab 2.5 mg versus l-glutamine: QAR 73,226								

**Table 4.** Probabilistic sensitivity analysis results with their uncertainty ranges.

Variable	Variation range
Crizanlizumab 5 mg	
Success without pain crisis	0.3888, 0.5152, 0.6401, 5, 95
AEs	0.7569, 0.86, 0.9357, 5, 95
Without AEs	0.0643, 0.14, 0.2431, 5, 95
Failure	0.3599, 0.4848, 0.6112, 5, 95
Failure due to first pain crisis	0.8126, 0.92, 0.9659, 5, 95
Failure due to ACS	0, 0, 0.05, 5, 95
Failure due to death	0.0037, 0.016, 0.1052, 5, 95
Second pain crisis	0.3599, 0.48, 0.6112, 5, 95
No second pain crisis	0.3888, 0.52, 0.6401, 5, 95
Additional pain crisis	0.0251, 0.07, 0.168, 5, 95
No additional pain crisis	0.832, 0.93, 0.9749, 5, 95
Crizanlizumab 2.5 mg	
Success without pain crisis	0.4637, 0.56, 0.7149, 5, 95
AEs	0.7685, 0.87, 0.9445,5, 95
Without AEs	0.0555, 0.13, 0.2315, 5, 95
Failure	0.3137, 0.4375, 0.5672, 5, 95
Failure due to first pain crisis	0.8476, 0.92, 0.9827, 5, 95
Failure due to ACS	0, 0, 0.05, 5, 95
Failure due to death	0.0038,0.0352, 0.1084, 5, 95
Second pain crisis	0.3137, 0.4375, 0.5672, 5, 95
No second pain crisis	0.4328, 0.57, 0.6863, 5, 95
Additional pain crisis	0.0891, 0.16, 0.2868, 5, 95
No additional pain crisis	0.7132, 0.84, 0.9109, 5, 95
l-Glutamine	
Success without pain crisis	0.4303, 0.4503, 0.6306, 5, 95
AEs	0.943, 0.98, 0.9959, 5, 95
Without AEs	0.0041, 0.02, 0.057, 5, 95
Failure	0.4667, 0.5497, 0.6306, 5, 95
Failure due to first pain crisis	0.7433, 0.81, 0.8731, 5, 95
Failure due to ACS	0.0466, 0.15, 0.1678, 5, 95
Failure due to death	0.0041, 0.02, 0.057, 5, 95
Second pain crisis	0.325, 0.41, 0.4868, 5, 95
No second pain crisis	0.5132, 0.6, 0.675, 5, 95
Additional pain crisis	0.0147, 0.04, 0.0845, 5, 95
No additional pain crisis	0.9155, 0.96, 0.9853, 5, 95

### Sensitivity analysis results

#### One-way sensitivity analysis

##### Crizanlizumab 5 mg versus l-glutamine

Cost inputs in one-way sensitivity analysis, and their uncertainty distributions, are presented in Table 5. The model was insensitive to changes in all cases.

**Table 5.** One-way sensitivity analysis on cost input, distributions—crizanlizumab 5 mg versus l-glutamine.

Unit cost of medications	Point estimate (QAR)	Uncertainty range (triangular)		ICER
		Lower	Upper	
Crizanlizumab 5	6678	5676	7680	Mean: 79,424, 95% CI (75,548–83,150)
l-Glutamine	96	82	110	

#### Crizanlizumab 2.5 mg versus l-glutamine

Cost inputs in one-way sensitivity analysis, and their uncertainty distributions, are presented in Table 6. The model was insensitive to changes in all cases.

**Table 6.** One-way sensitivity analysis on cost input, distributions—crizanlizumab 2.5 mg versus l-glutamine.

Unit cost of medications	Point estimate (QAR)	Uncertainty range (triangular)		ICER
		Lower	Upper	
Crizanlizumab 2.5	6,678	5676	7680	Mean: 73,226, 95% CI (69,010–78,147)
l-Glutamine	96	82	110	

#### Crizanlizumab 5 mg versus crizanlizumab 2.5 mg

Cost inputs in one-way sensitivity analysis, and their uncertainty distributions, are presented in Table 7. The model was insensitive to changes in all cases.

**Table 7.** One-way sensitivity analysis on cost input, distributions—crizanlizumab 5 mg versus crizanlizumab 2.5 mg.

Cost of medications	Point estimate (QAR)	Uncertainty range (triangular)		ICER
		Lower	Upper	
Crizanlizumab 2.5	6678	5676	7680	Dominance. Mean cost saving: 3552, 95% CI (3015–4145)
Crizanlizumab 5 mg	6678	5676	7680	

#### Probabilistic sensitivity analysis

**Figure 2.**
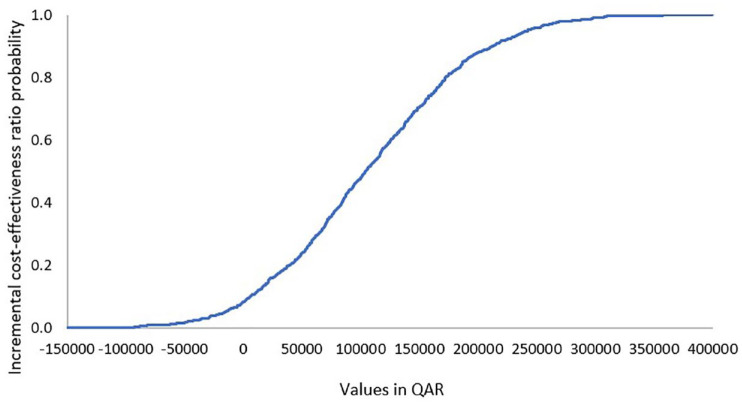
ICER acceptability curve of crizanlizumab 5 mg.

Model cost inputs and their plausible ranges are presented in Table 6. The ICER probability curve showed that crizanlizumab 5 mg was cost-effective in nearly 90% of simulated cases and was dominant in less than 10% of the cases (Figure 2).

**Figure 3.**
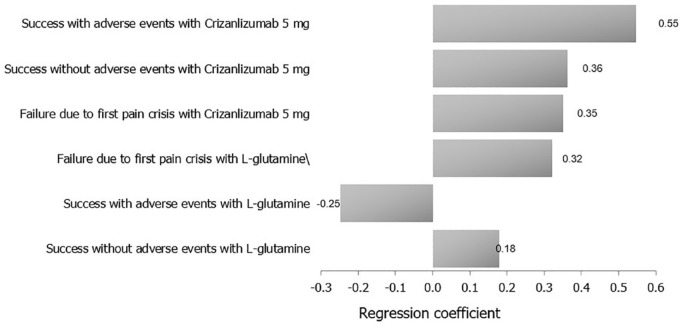
A tornado analysis of the study’s clinical outcomes and their costs on ICER (crizanlizumab 5 mg versus l-glutamine).

Based on the multivariate uncertainty analysis, a tornado regression analysis of the different study outcomes revealed that success with AEs with crizanlizumab 5 mg was the input that had the main effect on the outcome, while success without AEs with l-glutamine had the least effect on the outcome. A tornado regression of outcomes as per the regression coefficients of their impact on the study result is displayed in Figure 3.

**Figure 4.**
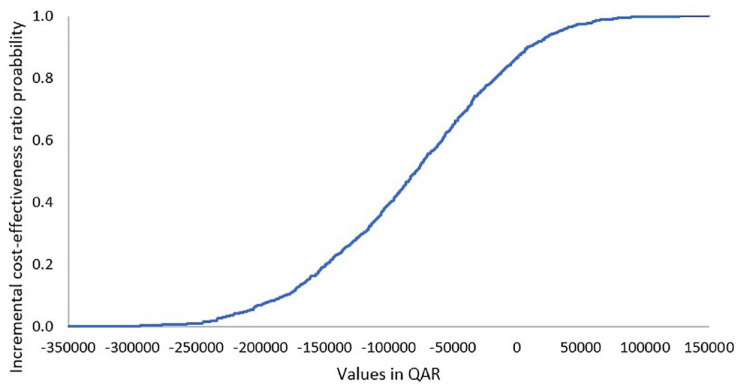
ICER acceptability curve of crizanlizumab 2.5 mg.

**Figure 5.**
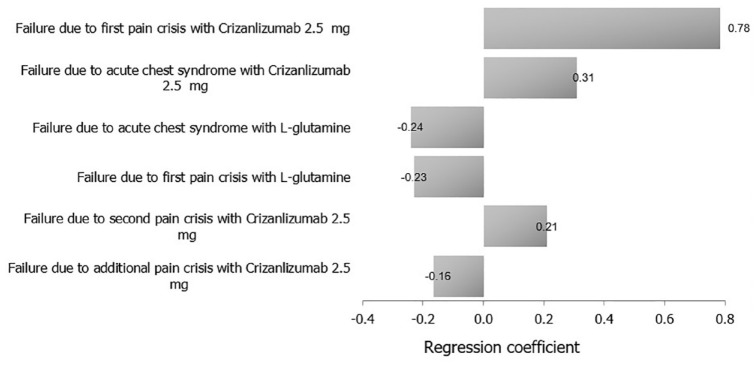
A tornado analysis of the study’s clinical outcomes and their costs on ICER (crizanlizumab 2.5 mg versus l-glutamine).

When comparing crizanlizumab 2.5 mg versus l-glutamine, model cost inputs and their ranges are presented in Table 6. The ICER probability curve showed that crizanlizumab 2.5 mg was cost-effective in nearly 80% of simulated cases (Figure 4). With regard to the tornado outcomes, failure due to first pain crisis with crizanlizumab 2.5 mg was the main influential factor, while failure due to additional pain crisis with crizanlizumab 2.5 mg was the least influential factor on the outcome (Figure 5).

**Figure 6.**
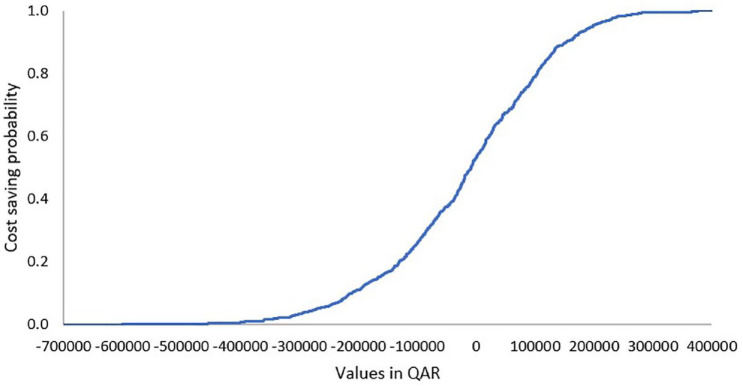
Cost saving acceptability curve of crizanlizumab 2.5 mg.

**Figure 7.**
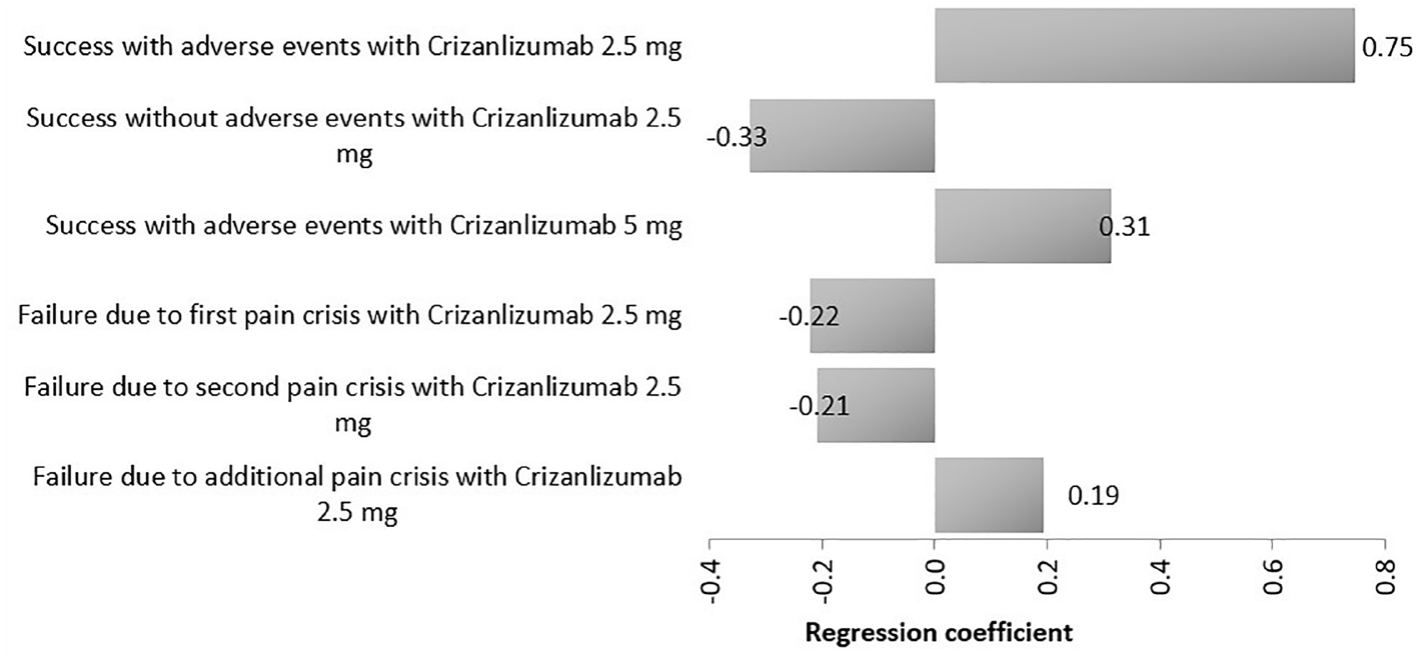
A tornado analysis of study medications costs on ICER (crizanlizumab 2.5 mg versus crizanlizumab 5 mg).

Finally, when comparing the two doses of crizanlizumab, model cost inputs and their ranges are presented in Table 6. Crizanlizumab 2.5 mg dominated the 5 mg regimen at the estimated WTP (Figure 6). Results of tornado diagram shows that success with and without AEs with crizanlizumab 2.5 mg were the key drivers of the outcome while failure due to additional pain crisis with crizanlizumab 2.5 mg was the least driver (Figure 7).

## Discussion

Based on previous systematic reviews and meta-analyses of SCD treatment modalities, hydroxyurea showed that it is effective in reducing VOC rates.^19,20^ Nevertheless, SCD patients who are receiving hydroxyurea can go on to have pain crises, subsequent organ damage, and higher mortality rate.^21^ Crizanlizumab and l-glutamine are newer FDA-approved options which are currently available in Qatar for SCD patients who may not be controlled with hydroxyurea. Nonetheless, no direct comparison between these treatments has been conducted, especially in the Middle East region.^13,22–24^

To the best of our knowledge, this is the first cost-effectiveness evaluation of crizanlizumab 5 mg, crizanlizumab 2.5 mg, versus l-glutamine for older adolescent and adult (⩾16 years old) SCD patients, and who are not or poorly controlled on hydroxyurea.

Our study, with a follow-up duration of a 1-year span, showed that the 5 mg crizanlizumab achieved a success rate of pain crisis averted of 0.5152 compared to 0.4503 with l-glutamine, with an ICER of QAR 79,424 ($21,814) with 5 mg crizanlizumab per patient. Also, 2.5 mg crizanlizumab achieved a success rate of pain crisis averted of 0.5625 compared to 0.4503 with l-glutamine, with an ICER of QAR 73,226 ($20,111) with 2.5 mg crizanlizumab per patient. The 5 mg crizanlizumab and 2.5 mg crizanlizumab achieved success rates of pain crisis averted of 0.515222/year and 0.29/year, respectively, with a cost saving of QAR 377,104 ($103,571) with crizanlizumab 2.5 mg over crizanlizumab 5 mg per patient. This is, therefore, a dominance (higher effectiveness and lower cost) in favor of crizanlizumab 2.5 mg over crizanlizumab 5 mg.

The sensitivity analyses confirmed the robustness of our findings and showed that the probability of success with AEs with crizanlizumab 5 mg had the main effect on the outcome in relation to the comparison between crizanlizumab 5 mg and l-glutamine, failure due to first pain crisis with crizanlizumab 2.5 mg was the main influential factor with regard to the comparison between crizanlizumab 2.5 mg and l-glutamine, and success rates with and without AEs with crizanlizumab 2.5 mg were the key drivers with regard to the comparison between crizanlizumab 5 mg versus crizanlizumab 2.5.

Of note, the acquisition cost of both drugs was the biggest cost driver in model analysis, followed by hospitalization costs.

While there are no similar literature models to compare to, our study model is focusing on the main outcome targeted for SCD management (i.e., VOC) followed, where the VOC averted was the outcome of interest since VOCs decrease the quality of life (QoL) and are the main cause of hospital visitations in SCD and the increase in the risk of death. It is essential to emphasize the model’s narrow focus, primarily centered on the single outcome of acute vaso-occlusive pain. It’s important to note that the available data did not afford us the opportunity to model other critical acute outcomes frequently associated with SCD, such as ACS, which stands as a leading cause of mortality in SCD. Additionally, the limitations of the data hindered us from modeling major chronic complications of SCD that carry substantial morbidity, economic burden, and a significant impact on the QoL, including conditions like stroke, chronic renal failure, and avascular necrosis. New therapies that reduce SCD hospitalizations are desirable given the potential to impact healthcare utilization, but also to reduce disease burden and decrease mortality and morbidity.^25,26^

## Limitations

Our economic evaluation of crizanlizumab and l-glutamine for the treatment of SCD in the context of Qatar has provided valuable insights. However, several important considerations should be taken into account when interpreting the results and implications of our analysis.

The presence of potential heterogeneity among patient subpopulations, particularly in a multinational country like Qatar, underscores the need for further investigation into diverse patient demographics, including factors such as race and ethnicity.

Additionally, our study is limited by the age restrictions of the RCT data sources, which may impact the generalizability of our findings to the pediatric SCD population. We anticipate that ongoing trials, such as the assessment of crizanlizumab in pediatric patients as young as 2 years of age, will provide valuable insights to enhance the accuracy of our economic model for this specific population.

While our study primarily focused on clinical and economic outcomes, we acknowledge the absence of an assessment of the QoL due to the lack of comparative QoL data from the RCTs. Moreover, the potential impact of drop-out rates and real-world medication compliance further emphasizes the need for cautious interpretation of our findings, emphasizing the importance of considering practical aspects of medication use beyond clinical efficacy.

It is important to acknowledge that our analysis has not incorporated potential additional benefits of these medications that have been presented in analyses such as Boshen Jiao et al.^27^ Furthermore, we recognize the need to clarify the model’s assumption regarding the reduction of ACS events with crizanlizumab. The report of zero ACS events in the pivotal trial by Ataga et al. does not necessarily indicate the complete elimination of ACS. Therefore, further investigation into the impact of crizanlizumab on ACS should be considered in future studies to refine the model’s assumptions.^28^

We also acknowledge the potential impact of glutamine on reducing blood transfusions, as indicated by Zaidi et al.^29^ The subjectivity and lack of uniformity in the decision to administer blood transfusions pose a challenge in assessing their precise impact. Addressing the variability in clinical practice and its implications on the economic evaluation is an important consideration for future studies.^29^

Furthermore, distinguishing between patients on and off hydroxyurea treatment is a valid concern. Addressing subgroups like pregnant women and those with renal failure, who may not be on hydroxyurea, should be a focus for future studies to comprehensively understand medication effectiveness and economic impact within these populations.^29^

Additionally, the limitations imposed by the availability of model inputs from RCT studies by Niihara et al. and Ataga et al. Nevertheless, it highlights the importance of incorporating a wider range of estimates in sensitivity analyses where feasible from other studies on both drugs.

Lastly, the differences in reporting criteria for adverse effects, as highlighted in the Niihara et al. and Ataga et al. studies, underline the need for future studies that standardize AEs reporting criteria. This standardization will enable meaningful comparisons and a comprehensive assessment of adverse effects and their associated costs.

## Conclusion

Our baseline analysis suggested that each of the 5 and 2.5 mg/kg doses of crizanlizumab reduces pain crises at a higher rate than l-glutamine in older adolescents and adults (⩾16 years old), with an acceptable relative AE profile, but at a higher cost. But, while both crizanlizumab doses were each cost-effective compared to the l-glutamine, the 2.5 mg/kg crizanlizumab was dominant over the 5 mg/kg crizanlizumab. This is in support of the recent trend of increasingly utilizing the 5 mg/kg crizanlizumab over l-glutamine in NCCCR, and if the unapproved use of the 2.5 mg/kg crizanlizumab is locally endorsed, this will further increase the efficiency of the crizanlizumab use. One limitation of our study must be noted, as our model’s primary focus is on acute vaso-occlusive pain. The available data did not allow for modeling other vital acute outcomes in SCD, such as ACS, a leading cause of mortality.
